# Remedies from nature: exploring the moderating mechanisms of natural landscape features on emotions and perceived restoration in urban parks

**DOI:** 10.3389/fpsyg.2024.1502240

**Published:** 2025-01-08

**Authors:** Yihe Li, Wenbo Li, Yang Liu

**Affiliations:** School of Art and Design, Zhejiang Sci-Tech University, Hangzhou, China

**Keywords:** urban park landscapes, emotional state, perceived restorativeness, natural landscape features, artificial landscape features, PLS-SEM

## Abstract

Urban parks are the primary places where urban residents reconnect with natural environments. Numerous studies have shown that natural landscape features benefit human mental health and promote perceived restoration. However, few studies have explored the extent to which natural landscape features in urban parks can mitigate or compensate for the negative effects of artificial landscape features on emotions and restoration. This study employed a field survey method, collecting questionnaire data from 599 participants in four urban parks in Hangzhou, China. The DeepLabV3+ semantic segmentation tool was employed to identify and extract landscape features from panoramic images. Data were statistically analyzed using Structural Equation Modeling (PLS-SEM) to explore the potential moderating effects of different natural landscape features in various environmental contexts on emotions and restoration. The results from the SEM model indicate that the R^2^ values for emotional state (ES) and perceived restorative scale (PRS) are 0.359 and 0.643, respectively, demonstrating an acceptable level of explanatory power and fit for the model. The results revealed that not all artificial landscape features negatively affect emotions and restoration. Although Pavement showed a significant negative impact on Perceived Restoration (*β* = −0.155, *p* = 0.004), their effect on emotions was not significant (*β* = 0.111, *p* = 0.115). Additionally, the study demonstrated that certain natural landscape features, such as the sky and trees, had a significant moderating effect in alleviating the negative emotions caused by artificial landscape features. However, for Perceived restoration, the moderating effect of these natural landscape features was not significant and, in some cases, even exhibited a negative moderating effect. These findings specifically explore how different natural landscape features can offset the adverse effects of artificial landscape features and, to varying degrees, enhance positive emotional responses and improve perceived restoration. The results contribute to understanding the complex dynamics between landscape features, emotions, and restoration in urban parks, offering strategic recommendations for planning, management, and design to create healthier and more restorative urban park environments.

## 1 Introduction

In the past 40 years, China has undergone a rapid and extensive urbanization process, with the urbanization rate increasing from 17.93% in 1978 to 58.52% in 2017 (Lei et al., 2023). The high-turnover, high-efficiency urbanization has, to some extent, turned urban spaces into hostile environments. The large population concentration has led to problems such as traffic congestion, housing shortages, and environmental pollution (Dong et al., 2021). In such high-density urban environments, residents have fewer opportunities to engage with nature, and the incidence of mental health issues such as anxiety, psychological disorders, and depression has significantly increased among urban dwellers (Luo and Jiang, 2022).

Urban parks, as important public open spaces, carry multiple functions and values within the urban environment. Studies have shown that during natural disasters, urban parks can serve as temporary shelters, providing a safe refuge for citizens (Shao et al., 2023). In addition, urban parks enhance residents’ quality of life and overall well-being by providing landscape services (Ma et al., 2024). They can effectively reduce negative emotions, reduce stress, and offer significant perceptual restorative benefits to individuals (de Vries et al., 2013; Markevych et al., 2017; Ishak et al., 2018). The physical environment of urban parks is composed of both natural landscape features (such as trees, shrubs, grassland, water, and the sky) and artificial landscape features (such as pavement and buildings) (Booth, 1989; Wang et al., 2016). The overall restorative benefits of these parks depend on a balanced control and refined design between the two (Aboufazeli et al., 2024). Different natural landscape features vary in their ability to improve emotions and promote restoration (Zhang et al., 2023; Shen et al., 2024). Therefore, it is important to thoroughly examine the varying effects of different natural landscape features on emotional regulation and restoration to mitigate the potential negative impacts of artificial landscape features (de Vries et al., 2003). This helps to fully realize the potential of urban parks in enhancing individuals’ emotions and restoration.

In environmental studies, emotions and affect are often used interchangeably (Frost et al., 2022), but they differ significantly in several key dimensions. Affect refers to a broader, longer-lasting state (Ekkekakis and Petruzzello, 2000). Emotions are typically immediate responses to specific stimuli, representing a conscious, context-related, and brief process influenced by particular objects (Beedie et al., 2005; Fiebig et al., 2020). The concept of emotions includes intense feelings such as anxiety, anger, and excitement, which can vary according to context and cognitive appraisal (Mulligan and Scherer, 2012). Additionally, as complex physiological and psychological states (Spielberger and Reheiser, 2009), emotions are regarded as a core mechanism for improving health (Ulrich et al., 1991). The research by Wilkie and Davinson (2021b) explores nature-based interventions (NBIs), a health promotion activity aimed at achieving restoration and recovery through contact with real or technologically simulated natural environments. These interventions help prevent emotional health issues by providing interaction with nature (Wilkie and Davinson, 2021a). Additionally, it is noteworthy that the condition restoration theory proposed by Egner et al. (2020) supports the role of emotions in the recovery process. This theory examines how natural environments conditionally affect individuals’ recovery processes, with studies finding that contact with natural environments has a more pronounced positive impact on emotions than on attention improvement. This enhancement of emotions may be an important pathway through which natural environments influence cognitive functions such as attention.

The theory of restorative environments is primarily guided by two theories: the Attention Restoration Theory (ART) proposed by the Kaplan and Kaplan (1989), and the Stress Recovery Theory (SRT) proposed by Roger Ulrich (Ulrich, 1979). Both theories suggest that exposure to environments with natural characteristics can help alleviate stress, improve emotions, and promote perceptual recovery. A number of studies support this view (Daniels et al., 2022; Li et al., 2023; Stragà et al., 2023), and Gross suggested that, in complex situations, individuals’ emotion regulation strategies also influencethe effectiveness of restorative environments in facilitating emotional recovery (Gross, 2015). Additionally, in restorative environment research, perceptual fluency suggests that natural environments, due to the concept of “fractals, “often exhibit greater coherence and fluency than urban environments (Kaplan and Kaplan, 1989). This fractal concept describes self-similar patterns repeated across different scales, such as branches that are smaller versions of the whole tree. This makes natural scenes easier to process visually and more restorative than urban or other built environments (Joye and van den Berg, 2011).

Based on these theoretical premises, current research generally acknowledges that natural landscape features have an advantage over artificial landscape features in improving emotional states and enhancing restoration (Bowler et al., 2010; Lindal and Hartig, 2015). Nordh et al. (2009) proposed design characteristics of “highly restorative environments” and “low-restorative environments.” Highly restorative environments include abundant natural features, such as lawns, vegetation, and water bodies, while low-restorative environments are often dominated by hard surfaces and transportation infrastructure (Nordh and Østby, 2013). Moreover, it is important to note that early studies by Ulrich et al. pointed out that in environments rich in natural features, the negative effects of artificial facilities may be offset by the positive effects of natural landscapes (Ulrich et al., 1991). This suggests that natural landscape features can, to some extent, intervene in the environment to mitigate or alleviate the negative impacts caused by artificial landscape features.

However, most existing studies focus on assessing the overall benefits of natural environments, primarily revealing the direct effects of natural landscape features on emotions and perceived restoration (Wu and Kim, 2021) or analyzing the benefits of individual natural or artificial landscape features. Few studies explore whether, within the complex context of urban parks—where diverse landscape features intertwine—landscape characteristics can influence individuals’ restoration through enhanced emotions. Furthermore, the specific mechanisms by which emotions function as a mediating variable in this process remain underexplored. Thus, a question worthy of in-depth investigation is whether the interaction between various natural and artificial landscape features in urban parks can elicit complex emotional responses of differing intensities, thereby further influencing perceived restorativeness in the environment. The absence of research in this area has led to limitations in providing comprehensive guidance for landscape planning and design in complex urban park settings. Addressing this research gap would allow for a more detailed analysis of the pathways through which natural landscapes contribute to perceived restoration, deepening our understanding of psychological restoration processes.

In conclusion, this study aims to discuss how natural landscape features mitigate the negative effects of artificial landscape features on emotions and restoration, and to explore the pathway mechanisms between natural landscape features, emotions, and restoration. Given the complex interactions between multiple latent variables in the study, the research hypotheses are developed and validated using Partial Least Squares Structural Equation Modeling (PLS-SEM). As an advanced structural equation modeling technique, PLS-SEM is more effective than traditional regression analysis in handling the relationships between multiple variables, while providing comprehensive estimates for both measurement and structural models (Ali et al., 2018). The study was conducted in four urban parks in Hangzhou, China, where data was collected through field surveys and questionnaires. Additionally, many high-resolution landscape images were collected, and the DeepLabV3+ semantic segmentation tool based on deep learning technology was used to classify and extract the landscape features from the images. This comprehensive approach enables the study to reveal the synergistic or offsetting effects between various landscape features, providing more concrete evidence to support the design and planning of urban parks. It helps designers and planners create urban park spaces with greater restorative potential.

## 2 Literature review

### 2.1 The influence of landscape features on perceived restoration

The Attention Restoration Theory (ART) and the Stress Recovery Theory (SRT) both explain the restorative effects of natural environments. According to ART, prolonged directed attention can lead to fatigue, while natural environments alleviate attentional fatigue by engaging involuntary attention, thus restoring cognitive function (Kaplan and Kaplan, 1989). Environments that facilitate recovery from directed attention fatigue are referred to as restorative environments (Kaplan, 1995). In contrast, SRT emphasizes that exposure to nature can rapidly alleviate stress in the short term, lowering blood pressure and cortisol levels, with a stronger focus on psychological and physiological recovery (Ulrich, 1986; Ulrich et al., 1991). Both theories suggest that natural environments can relieve stress and enhance emotion, thereby promoting recovery (van den Berg et al., 2003; Stragà et al., 2023). The Perceived Restorativeness Scale (PRS), created by Hartig et al. (1996), is grounded in Attention Restoration Theory (ART) and is designed to measure environments that engage involuntary attention. This scale serves as a tool for assessing the positive impact of stimulating environmental factors on fatigue recovery. The PRS evaluates the restorative potential of both current and proposed environments by evaluating four key characteristics: being away, fascination, compatibility, and coherence (Tenngart Ivarsson and Hagerhall, 2008). “Being away” refers to the sense of escaping from fatigue or stress, evaluating the feeling of rest and recovery from mental exhaustion when individuals perceive themselves as detached from daily life in a specific environment. “Fascination” indicates that the environment should be sufficiently interesting to capture and hold people’s attention, making them want to engage more with it. Natural landscapes, with their great potential to create moments of fascination, are therefore seen as having a strong ability to combat mental fatigue. “Compatibility” refers to how well the environment aligns with individual preferences, goals, and needs, allowing visitors to engage in activities without restrictions. “Coherence” implies that a space should contain enough features to make visitors feel as though they are moving away from stress. The Perceived Restorativeness Scale (PRS), grounded in ART, has proven effective in assessing the psychological recovery benefits of environments (Bornioli and Subiza-Pérez, 2023). Many studies have used the PRS to evaluate restorative environments and restorative landscape features in urban settings (Carrus et al., 2015; Liu et al., 2020; Hooyberg et al., 2022).

Currently, the restorative effects of natural environments are widely recognized (Pouso et al., 2020; Li et al., 2023), and research has revealed the role of various landscape features in perceived restorativeness. Studies by Hartig et al. (2003) indicate that natural and urban environments have different impacts on attention restoration, suggesting that providing accessible natural landscapes for urban residents may help prevent and reduce stress-related health issues. Jiang et al. (2014) found that green vegetation significantly reduces anxiety and stress levels, enhancing perceived restorativeness, while artificial landscape features such as roads and buildings are closely associated with decreased restorativeness (Wang et al., 2016). Additionally, a laboratory test evaluated the restorative benefits of different environmental elements, such as plants and water, in 12 green spaces. The results showed that these landscape features have restorative effects, with semi-open green spaces high in naturalness offering more positive restorative outcomes than open green spaces with higher levels of artificiality (Liu et al., 2022). Similarly, some international studies have also confirmed the general restorative benefits of natural elements. For example, Akpınar (2021) research found a universally positive impact of nature on perceived restoration among adolescents in Turkey, regardless of gender. In summary, existing research has primarily focused on comparing the overall restorative effects of natural versus non-natural environments or examining the general relationship between landscape features and perceived restorativeness by comparing the restorative effects of different landscape features. However, there is still a lack of exploration regarding how specific elements in natural environments stimulate restorative potential in different contexts. Few studies have delved into the specific ways in which landscape features function to influence the restoration process.

### 2.2 The influence of landscape features on emotion

Currently, enhancing residents’ positive emotions has become a fundamental requirement for achieving the United Nations Sustainable Development Goals of “Good Health and Well-being” and “Sustainable Cities and Communities” (dpicampaigns, n.d.). Ulrich’s theory of stress recovery indicates that emotions are regarded as a core mechanism for health improvement (Ulrich et al., 1991). Natural environments can reduce physiological stress responses by activating the parasympathetic nervous system, thereby lowering anxiety and improving emotions (Wilkie and Davinson, 2021a). However, with the rapid pace of urbanization, the expansion of artificial landscapes has intensified residents’ feelings of anxiety, becoming a significant factor affecting the emotional health of urban residents (de Vries et al., 2003). Many studies have emphasized the different emotional impacts of natural and artificial landscapes, showing that people can experience varying degrees of emotional healing from natural environments (Zhang, 2023). In this regard, urban park environments have been proven to effectively alleviate visual fatigue, reduce negative emotions (Berto, 2014), and enhance the well-being of both residents and visitors (Rahnema et al., 2019). For instance, the study conducted by Wilkie et al. (2022) compared the effects of different types of environments on individuals’ emotions, mood, and perceived restoration. The findings indicate that even brief exposure to virtual environments can elicit perceptual differences regarding environmental restoration. Moreover, natural environments and urban green spaces were found to generate stronger positive emotions than urban streets, while the impact on mood was less pronounced (Wilkie et al., 2022). Particularly during the COVID-19 pandemic, a substantial body of evidence suggests that time spent in natural environments can reduce stress and enhance mental well-being. Residents who live in proximity to urban parks are less prone to experiencing negative emotions (Ma et al., 2022). And after walking in a natural environment, it may bring better emotional recovery benefits (Hartig et al., 2003). Moreover, numerous empirical studies have further highlighted the impact of landscape features in natural environments on emotional states. For example, natural features such as green spaces and water bodies have been shown to benefit the emotional health of urban residents (Lu et al., 2021; Wu and Kim, 2021), thereby alleviating the stress of modern urban life (Joye, 2007). Tsai et al. found a significant correlation between shrub coverage and the incidence of psychological distress (Tsai et al., 2018). Olszewska-Guizzo et al. (2022) explored the connection between urban green spaces and positive emotions and relaxation, highlighting the impact of landscape visual quality on emotional experiences. These studies emphasize the direct impact of natural environments and their features on emotional health, helping to lower the risk of depression and anxiety (Wu et al., 2023). This aligns with Ulrich’s Stress Recovery Theory (SRT), which suggests that exposure to or viewing vegetation and other natural features can quickly have a positive impact on individuals experiencing acute stress, thus helping to avoid negative thoughts and emotions (Ulrich et al., 1991).

Although numerous studies have highlighted the positive impact of natural environments and landscape elements on emotional states (Collins et al., 2020), the pathway mechanisms by which natural landscape elements influence perceived restoration through emotional impact have yet to be thoroughly explored. Further research is needed to investigate how the moderating potential of natural landscape elements can indirectly affect perceived restoration through emotions, which would contribute to the sustainable environmental design of urban parks for health and well-being.

### 2.3 Hypothesis

Although existing research has widely confirmed the differential effects of natural and artificial landscape features on emotions and restorative outcomes, these studies have mostly focused on the restorative effects of individual landscape features, often overlooking the complex moderating roles between different landscape characteristics in influencing emotions and restoration. This may result in the underutilization of the restorative potential within urban park environments. While many studies indicate that artificial landscape features generally lack the restorative benefits of natural landscapes and can negatively impact emotions and restoration, Attention Restoration Theory (ART) and Stress Recovery Theory (SRT) suggest that, with thoughtful design and layout, artificial features may promote recovery by enhancing environmental structure and order. This can support emotional restoration and reduce cognitive load, thereby fostering overall restoration. Therefore, although the restorative capacity of built landscape features differs from that of natural landscape features, the moderating effect of natural elements may counterbalance the negative impact of built features, enhancing the overall restorative support of the environment. Additionally, as a widely used mediating variable, the enhancement of emotions may be a key pathway through which natural environments promote restorative effects; the positive emotions generated by the environment may indirectly support cognitive function and attention recovery. Thus, identifying the relationship between built landscape features and emotional recovery, as well as exploring the moderating effect of natural landscape features on the negative impact of built features, can offer a more comprehensive understanding of how landscape characteristics influence restorative processes. This approach enables more effective design and placement of landscape elements in urban parks.

**Figure 1:**
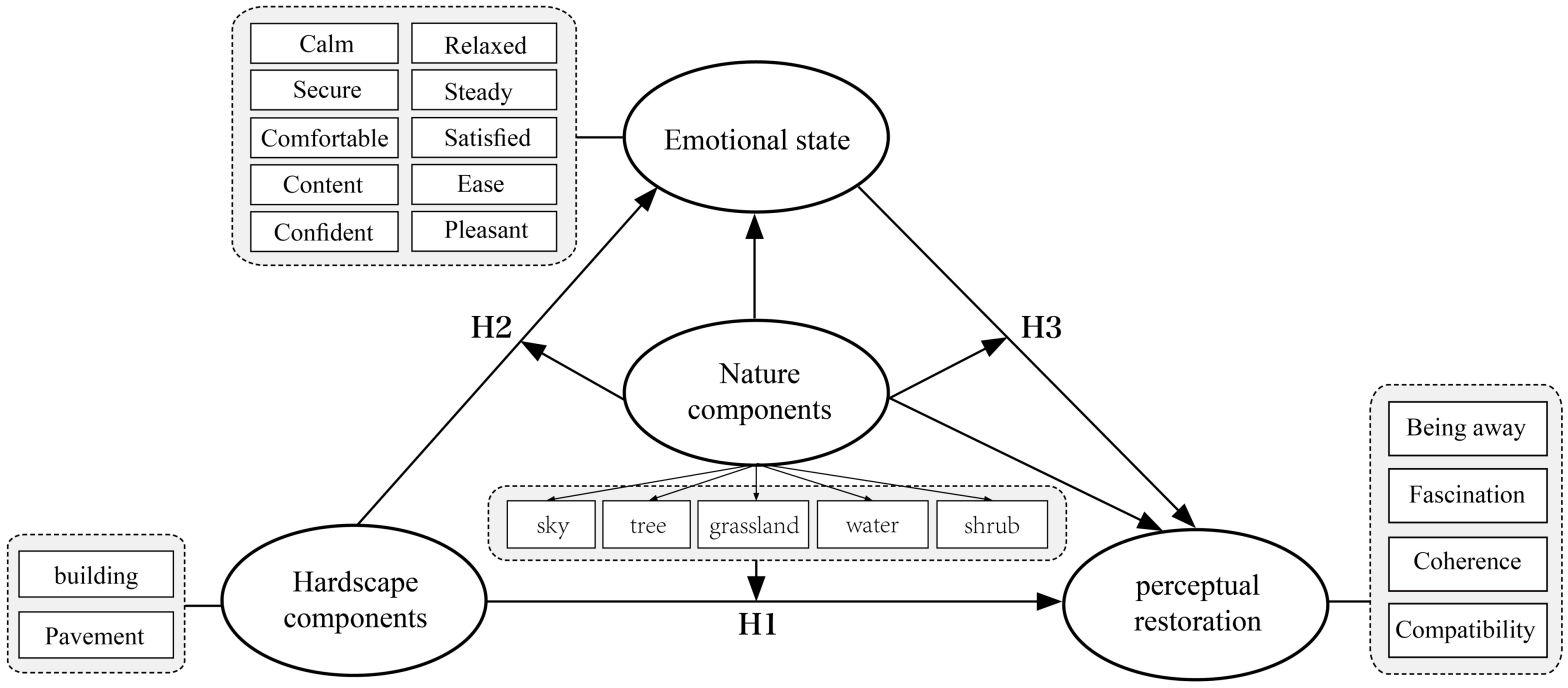
A mediation model framework with multiple factors moderation.

Based on this, this study constructs a moderated mediation model (Figure 1). In this model, emotion serves as the mediating variable, while natural landscape features act as the moderating variable. The statistical technique employed to test the hypotheses in this study is Partial Least Squares Structural Equation Modeling (PLS-SEM). Being a predictive method, PLS-SEM can simultaneously estimate the interrelationships among multiple variables and help validate the hypotheses to explore the pathway mechanisms between variables. The following are the three research hypotheses proposed:

*H1:* Artificial landscape features in urban parks (buildings, pavement) indirectly negatively affect perceived restoration by influencing emotions.

*H2:* Natural landscape features in urban parks (sky, trees, pavement, water, shrubs) moderate the negative effects of artificial landscape features (buildings, roads) on emotions.

*H3:* Natural landscape features in urban parks (sky, trees, grass, water, shrubs) moderate the negative effects of artificial landscape features (buildings, roads) on perceived restoration.

## 3 Methods

### 3.1 Study sites

This study was conducted in Hangzhou, Zhejiang Province, China. As a city in southeastern China, Hangzhou is renowned for its scenic resources and historical culture, attracting many visitors. According to statistics, Hangzhou had a permanent population of 12.52 million in 2023, with an annual passenger traffic of 160 million. Hangzhou’s urban parks have become important venues for residents and tourists to engage in leisure and cultural tourism activities. In the past decade, the city has undergone rapid economic growth and significant land use changes during urban expansion. From 2017 to 2021, the area of urban green spaces increased, with most of the growth concentrated in the city’s core areas (Chen et al., 2021). Concurrently, the ‘Hangzhou Green Space System Special Plan (2021–2035)’ sets goals for 2035, which include guaranteeing that the per capita green park space is no less than 17 square meters, the service radius coverage of park green spaces reaches at least 95%, and the green coverage rate is maintained at no less than 43% [Hangzhou green space system special plan (until 2035) released, 2024]. This highlights Hangzhou’s commitment to green space planning.

**Figure 2:**
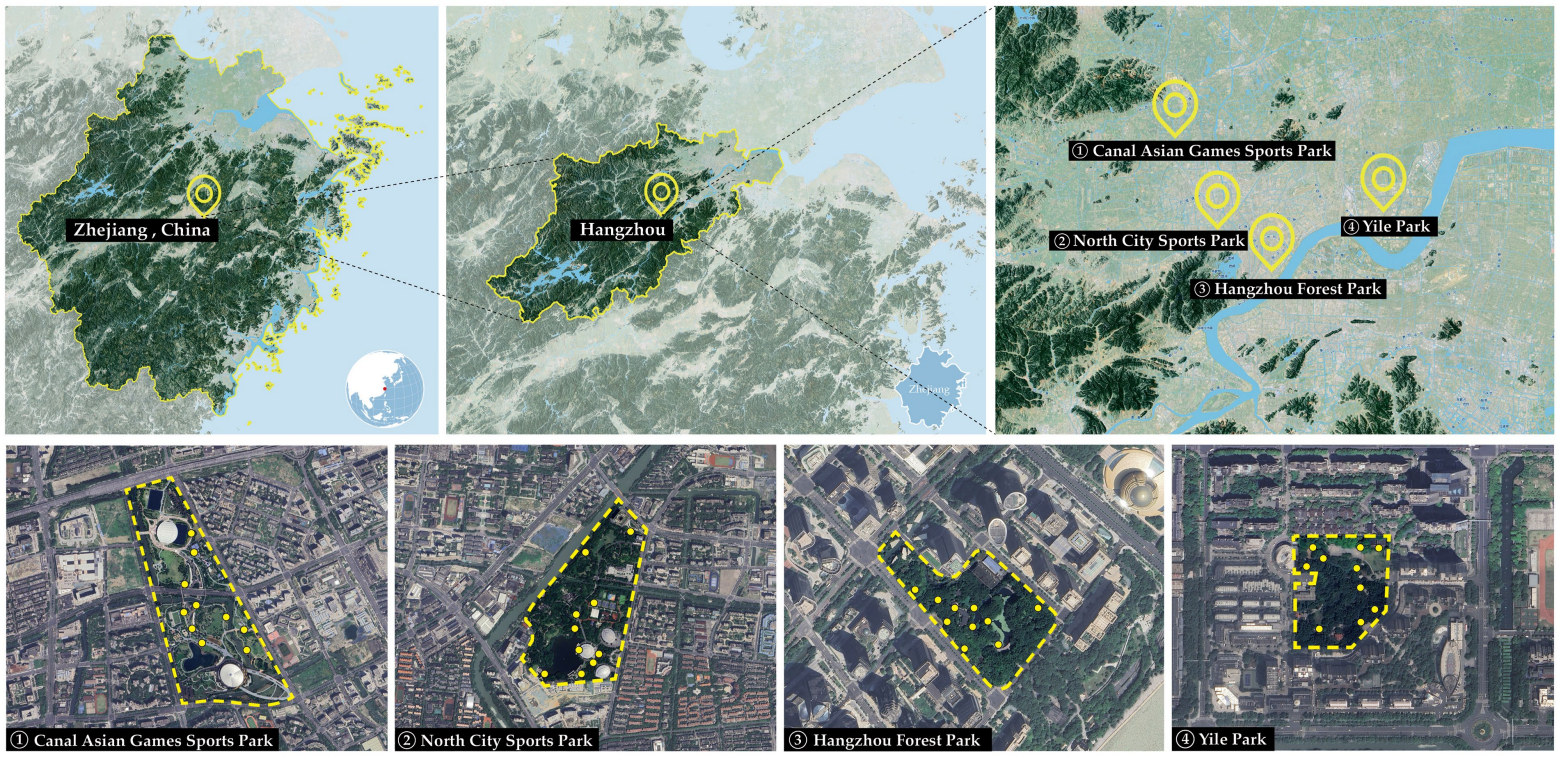
Study site areas.

The selection of suitable parks as research sites was based primarily on two criteria. First, the selected parks must be open to the public for free to attract a diverse range of participants. Second, the landscape environment within the chosen parks should be representative, containing typical landscape features commonly found in parks, to ensure the study’s applicability. Based on these criteria, four parks in the core urban area of Hangzhou (Canal Asian Games Sports Park, North City Sports Park, Hangzhou Forest Park, and Yile Park) were ultimately selected for field surveys (Figure 2). In each park, researchers selected 10 locations with typical landscape features for assessment. The Canal Asian Games Park is in Gongshu District, Hangzhou, with a total area of 46.7 hectares. It is a comprehensive urban sports park featuring a variety of relaxation spaces, sports areas, and outdoor landscapes. There is a rich variety of tree and flowering plant species, with the park preserving two original natural rivers and adding an artificial lake and wetlands. North City Sports Park, also located in Gongshu District, covers an area of 45 hectares and integrates sports, ecology, leisure, and entertainment into one sports park. The park includes a 3.8 hectare artificial lake, with green space covering over 86% of the area. Hangzhou Forest Park, located in Shangcheng District, covers 5.5 hectares, and is situated at the heart of the Qianjiang New City Central Business District (CBD). The park mainly serves as a place of leisure and social interaction for office workers and nearby residents. The park layout centers around a natural ecological water system, with an extensive road network, and features tall trees such as camphor and cedar. Yile Park is in Hangzhou’s Qiantang District, with a total area of 5.1 hectares. Surrounded by residential communities, it has become an ideal location for residents’ daily leisure, social, and family activities. The park features an ecological riverbank, with vegetation primarily consisting of willows and various flowering trees and shrubs.

### 3.2 Landscape features data collection

**Figure 3:**
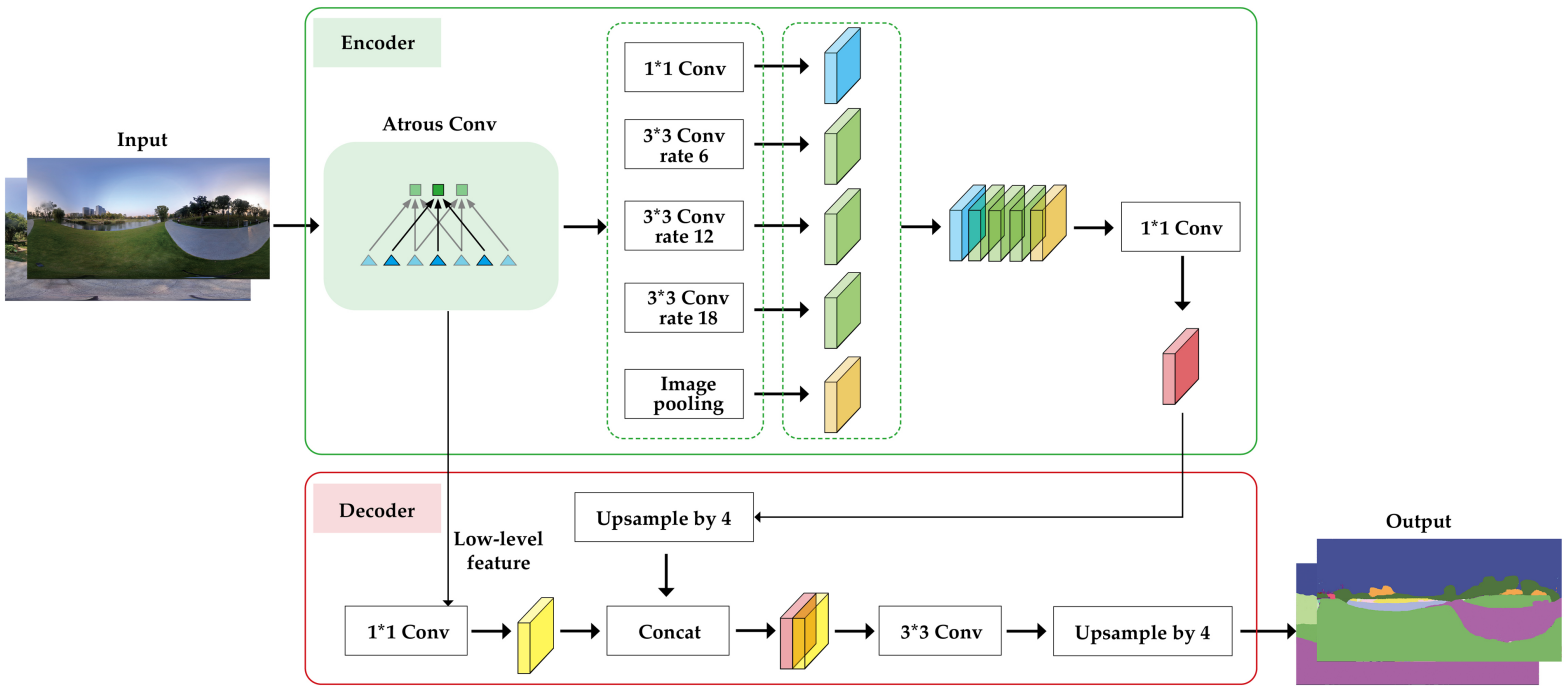
The structure of DeepLabV3+ algorithm.

To collect data on landscape features in the parks, this study conducted photo collection alongside the questionnaire survey. The questionnaires and photos were collected under consistent timing and conditions, from September to November 2023, between 9: 00 a.m. and 6:00 p.m. On days with clear skies and no rain. During this period, temperatures ranged from 20°C to 31°C, with wind speeds between 1 and 4 m/s, and humidity levels fluctuating between 51 and 98%. These specific weather conditions were sourced from the China Meteorological Data Service Center (China Meteorological Data Network, 2023). First, at the selected locations, panoramic static images were captured on-site using an Insta 360° One X camera. The camera has a resolution of 6080*3040 and is equipped with two F2.0 fisheye lenses. The camera height was set at 1.6 meters (Wang et al., 2016). Due to differences in landscape features across various scenes, manually identifying and extracting the areas of landscape features is a challenging task. However, Convolutional Neural Networks (CNN) have demonstrated outstanding advantages in visual recognition tasks (Sun et al., 2020). As an enhanced third-generation iteration of the DeepLab neural network, DeepLabV3+ has accelerated the network training speed and improved the accuracy of image feature extraction compared to the previous V1 and V2 versions (Gou et al., 2022). Therefore, to address this challenge, the study used the semantic segmentation tool DeepLabV3+, based on a deep convolutional neural network, to process the panoramic images. Through this method, the visual landscape features in the images were segmented into different categories, with the pixels of each category converted into uniform colors, thus determining the share of each landscape feature in the image for each location (Figure 3). Finally, based on the landscape element proportions returned by DeepLabV3+, we selected six common landscape features—buildings, roads, sky, trees, grass, water, and shrubs—for subsequent statistical analysis.

### 3.3 Assessment of emotional state and perceived restoration

From September to November 2023, we carried out an on-site intercept survey across four urban parks in Hangzhou, collecting a total of 599 valid questionnaires. The target population included frequent park-goers and city residents. The survey was conducted by trained graduate students, who gathered paper-based questionnaires through in-person interactions. Participants were randomly chosen from different locations within the parks. To capture the primary activity times of park visitors, data collection was conducted from 9: 00 AM to 6:00 PM. The questionnaire used in this study consists of two sections: an emotion assessment (Table 1) and a perceived restorative assessment (Table 2). First, the emotion assessment section refers to the State–Trait Anxiety Inventory (STAI) scale employed in a 2023 study by Wang Y. et al. (2023), specifically the State Anxiety Inventory (SAI) part of STAI, which includes ten positive psychological states. The STAI scale is composed of two parts: SAI, which measures anxiety states in temporary situations, and TAI, which measures chronic anxiety levels over an extended period (Spielberger et al., 2017). Numerous studies have found that STAI effectively measures broader negative emotions in practical applications, beyond just anxiety (Bados et al., 2010). According to Ekman’s research, emotions, as one of the basic psychological processes, include intense feelings like anxiety and anger and consist of multiple sub-emotions. For instance, within the positive emotion spectrum, forms like excitement, relief, and contentment together form core emotional states (Ekman, 1992). Furthermore, Scherer’s Component Process Model (CPM) posits that emotions are multidimensional and interdependent processes (Scherer, 2005). Given the immediacy, multidimensionality, and context-dependency of emotions, the SAI scale is effective in capturing momentary emotions triggered by environmental stimuli, such as those encountered in green spaces or public areas, thereby reflecting the emotional state of visitors. The questionnaire ranges from (1) absolutely not, (2) no, (3) not true, (4) somewhat, (5) a lot, to (6) very much. Higher scores indicate lower immediate anxiety levels and higher positive emotions (Zsido et al., 2020). For the assessment of perceived restoration, the study referenced the Perceived Restoration Scale (PRS) developed by Hartig et al. (1997)), along with additional modifications made by Zhao et al. (2023). The scale used in this study includes 20 items to measure four restorative dimensions (Being Away, Fascination, Compatibility, and Coherence). These items are evaluated using a Likert scale ranging from 1 = “completely disagree” to 7 = “completely agree.”

**Table 1:** State–trait anxiety inventory.

Construct	Operational definition	Item	Reference
ES	/	I feel calm. I feel secure. I feel comfortable. I feel content. I feel self-confident. I feel relaxed. I feel steady. I feel satisfied. I feel at ease. I feel pleasant	Wang Y. et al. (2023)

**Table 2:** Perceived restoration scale.

Dimensions	Item	Reference
Being-Away	Q1: Being here is an escape experience. Q2: Spending time here gives me a break from my day-to-day routine. Q3: It is a place to get away from it all. Q4: Being here helps me to relax my focus on getting things done. Q5: Coming here helps me to get relief from unwanted demands on my attention.	Hartig et al. (1997)
Fascination	Q6: This place has fascinating qualities. Q7: My attention is drawn to many interesting things. Q8: I want to get to know this place better. Q9: There is much to explore and discover here. Q10: I want to spend more time looking at the surroundings. Q11: This place is boring. (Reversed item) Q12: The setting is fascinating.	
Compatibility	Q13: Being here is consistent with my personal desires. Q14: I can do things I like here. Q15: I have a sense that I belong here. Q16: I can find ways to enjoy myself here. Q17: I have a sense of oneness with this setting.	
Coherence (Extent)	Q18: This is a place where everything has its place. Q19: There’s so much to explore and discover in this place (enough to explore in all directions) Q20: This place is very spacious, with no barriers to obstruct my movement.	Zhao et al. (2023)

To ensure that the questionnaire, once translated into Chinese, could be accurately understood by respondents, the study invited two professionals fluent in English to conduct a rigorous and detailed translation of the State–Trait Anxiety Inventory (STAI) and the Perceived Restoration Scale (PRS) used in the study. Subsequently, we randomly selected 25 respondents in Hangzhou Forest Park for a pilot test to verify the comprehensibility of the questionnaire content. After confirming that all items in the Chinese version of the questionnaire were well understood, we conducted a Cronbach’s reliability analysis on the pilot test data. The results showed good internal consistency across dimensions (*STAI_Cronbach’s α_ = 0.748, PRS_Cronbach’s α_ = 0.776–0.879*).

After completing the pre-testing steps, we officially began the survey. The survey was conducted by three graduate students across four parks selected by the research team. Prior to the survey, the researchers obtained written informed consent from each participant, providing a detailed explanation of the survey’s purpose and procedures. Respondents indicated that they had read and understood the general information about the survey, indicated that they were volunteering to participate, and were aware that they had the right to discontinue their participation and revoke their consent at any time without any repercussions. Respondents were required to meet the following criteria: (1) self-assessed normal vision or corrected to normal levels, (2) verbally confirmed no psychological disorders, and (3) stayed in the target area for at least 5 min before data collection. These conditions were confirmed through face-to-face verbal questioning. Eligible respondents who agreed to participate were then able to begin filling out the questionnaire. The entire process took approximately 5–7 min. As a gesture of gratitude, participants who completed the questionnaire were given a small gift upon concluding the survey.

### 3.4 Analysis of data

In the preliminary data analysis phase, IBM SPSS Statistics Version 27 was used to analyze the subjective measurement data obtained from the questionnaire, and subsequently structural equation modeling was constructed using Smart PLS version 3.0. Partial least squares structural equation modeling (PLS-SEM) was chosen for estimating complex models with numerous constructs, indicators, and structural paths. This method is suitable for predictive and exploratory research, particularly when working with complex models that include multiple latent variables and pathways (Hair et al., 2019). Given the model complexity in the hypotheses of this study, PLS-SEM was applied to analyze the research model and clarify path analysis. For the evaluation of the measurement model, construct validity was assessed using Cronbach’s *α* and rho A, while convergent validity was verified through standardized factor loadings and average variance extracted (AVE). Discriminant validity was assessed using the Fornell-Larcker criterion, which stipulates that the square root of each construct’s AVE should be greater than its correlations with other constructs. Finally, discriminant validity was further verified using the heterotrait-monotrait (HTMT) criterion (Hair et al., 2012). To evaluate the structural model, the significance of path coefficients and model fit were examined using 5,000 bootstrapped samples to assess statistical significance. Lastly, multigroup analysis (MGA) was conducted to explore demographic differences.

## 4 Analysis result

### 4.1 Demographic information

**Figure 4:**
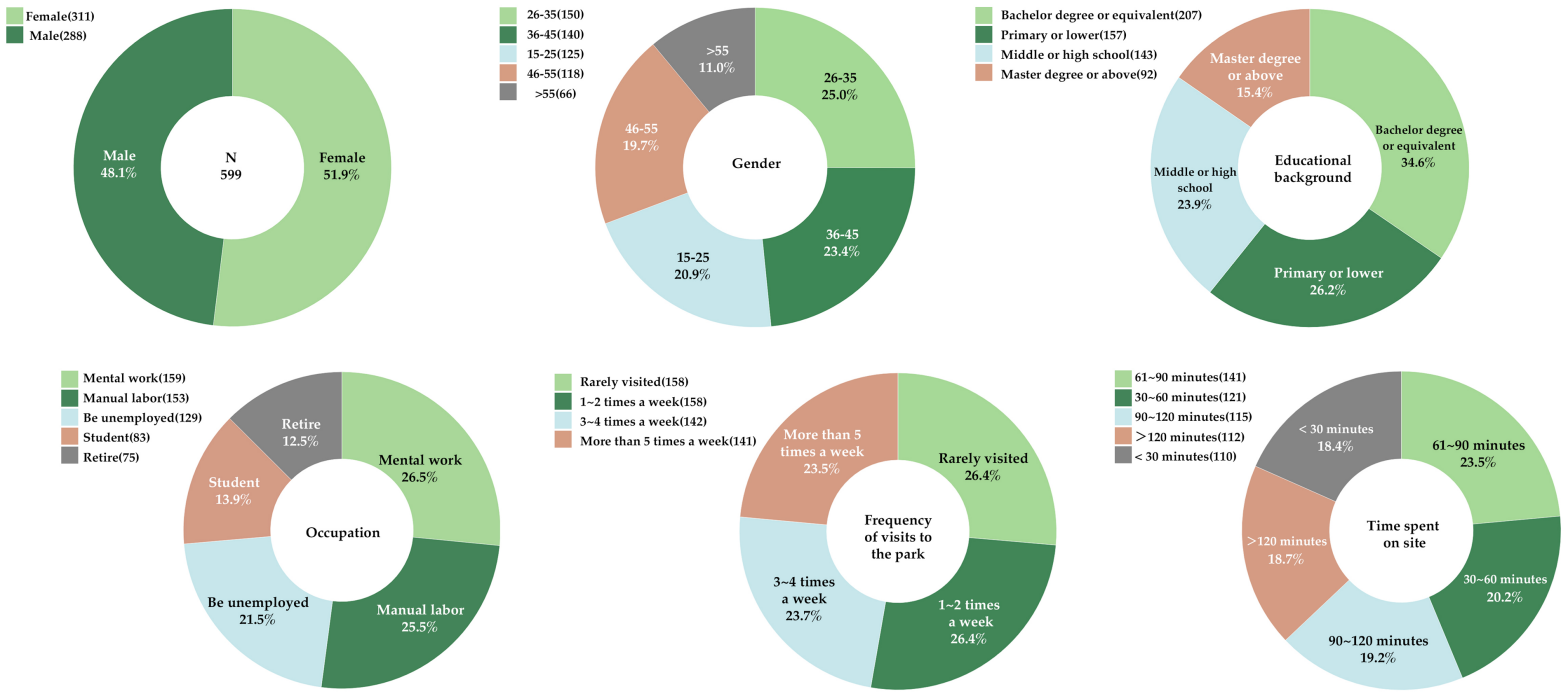
Demographic Information Chart.

According to Figure 4, respondents provided demographic details such as gender (male or female), age (15–25 years, 26–35 years, 36–45 years, 46–55 years, and above 55 years), educational background (primary school, middle school or below, high school, college or equivalent, master’s degree or above), and occupation (student, manual laborer, knowledge worker, retiree, unemployed). To assess the impact of green space exposure frequency and duration on residents perceived restorative effects, we asked about their park visit frequency (rarely, 1–2 times a week, 3–4 times a week, more than 5 times a week) and the duration of each visit (<30 min, 30–60 min, 61–90 min, 90–120 min, and > 120 min). A total of 611 questionnaires were sent out, and 599 responses were received, resulting in a 98% response rate. Among them, 150 responses were from Chengbei Sports Park, 150 from Hangzhou Asian Games Sports Park, 150 from Yile Park, and 149 from Hangzhou Forest Park.

### 4.2 Measurement model

The measurement model was evaluated to confirm the construct validity of Emotional State (ES) and the Perceived Restoration Scale (PRS). To achieve this, it is essential to meet several criteria, such as construct validity, discriminant validity, and convergent validity (Table 3). Additionally, to seek less biased and more consistent estimates, the study constructed a second-order structure for PRS. Construct validity was measured using Cronbach’s *α* and rho_A. The results showed that Cronbach’s *α* (0.775–0.921) and rho_A (0.782–0.912) for the substructures were above the recommended threshold of 0.70, indicating that the measurement model is reliable. For convergent validity, the results showed that the standardized factor loadings for all items were above the threshold of 0.5. Additionally, since the Average Variance Extracted (AVE) for both the first order and second-order structures ranged from 0.523 to 0.729, exceeding the required threshold of 0.5, the model’s convergent validity was confirmed. The study then integrated the latent scores of the four dimensions obtained in the first phase: Fascination (PRS_Fa), Being Away (PRS_BA), Compatibility (PRS_Comp), and Extent (PRS_Ext). The results indicated that the second-order factor loadings for these four dimensions ranged from 0.856 to 0.944, all above 0.7. The AVE was 0.849, Cronbach’s α was 0.94, rho_A was 0.949, and the VIF ranged from 2.531 to 4.985, indicating that there was no issue with multicollinearity. Table 4 presents the discriminant validity based on the Fornell-Larcker criterion. To establish discriminant validity, this criterion requires that the square root of the AVE for each construct must exceed its correlation coefficient with other constructs (Fornell and Larcker, 1981). In our analysis results, the values on the main diagonal of the correlation matrix were all higher than the off-diagonal features in the same row and column. For example, the highest correlation coefficient in the PRS_BA construct was 0.97, higher than its correlation with other constructs (ES = 0.485, PRS_Com*p* = 0.461), indicating that the measurement model has discriminant validity. Finally, the Heterotrait-Monotrait ratio (HTMT) criterion was used as a double-check to assess discriminant validity (Hair et al., 2012). The results in Table 5 show that the HTMT values were all below 0.85, confirming the establishment of discriminant validity. In conclusion, the checks for reliability and validity demonstrate that the measurement model has good adaptability.

**Table 3:** Reflective scale accuracy analyses (Summary of construct validity).

Reflective Components / Constructs and Items	Convergence validity		Consistency reliability		
	Factor loading	AVE	Cronbach’s α	rho_A	VIF
	>0.5	>0.5	>0.6	>0.6	<5
Step I: The first-order reflective components and unidimensional constructs were assessed.					
Perceived restorativeness (PRS)					
PRS_Fascination (PRS_Fa)					
PRS _Fa1	0.789	0.653	0.921	0.912	2.22
PRS _Fa2	0.745				2.292
PRS _Fa3	0.781				2.245
PRS _Fa4	0.774				2.436
PRS _Fa5	0.78				2.164
PRS _Fa6	0.777				2.356
PRS _Fa7	0.752				2.221
PRS _Being-Away (PRS_BA)					
PRS _BA1	0.785	0.729	0.907	0.907	2.257
PRS _BA2	0.804				2.424
PRS _BA3	0.821				2.55
PRS _BA4	0.801				2.731
PRS _BA5	0.816				2.896
PRS _Compatibility (PRS_comp)					
PRS _Comp1	0.788	0.72	0.903	0.903	2.575
PRS _Comp2	0.841				3.164
PRS _Comp3	0.806				2.461
PRS _Comp4	0.789				2.585
PRS _Comp5	0.788				2.19
PRS _Extent (PRS_Ext)					
PRS _Ext1	0.613	0.689	0.775	0.782	1.748
PRS _Ext2	0.682				1.666
PRS _Ext3	0.752				1.554
2. Emotional state (ES)					
ES_A	0.736	0.523	0.898	0.901	2.076
ES_B	0.763				2.072
ES_C	0.791				2.238
ES_D	0.744				1.954
ES_E	0.744				2.031
ES_F	0.745				1.856
ES_G	0.649				1.652
ES_H	0.679				1.762
ES_I	0.694				1.724
ES_J	0.677				1.692
Step II: The second-order reflective components.					
Perceived restorativeness (PRS)					
PRS_Fa	0.944	0.849	0.94	0.949	4.985
PRS_BA	0.941				4.254
PRS_Comp	0.942				4.489
PRS_Ext	0.856				2.531

**Table 4:** Discriminant validity assessment: Fornell-Larcker.

	Es	PRS BA	PRS Comp	PRS Ext	PRS Fa
Es					
PRS	0.466				
PRS BA	0.485				
PRS Comp	0.461	0.97			
PRS Ext	0.433	0.86	0.846		
PRS Fa	0.428	0.93	0.944	0.9	

**Table 5:** Discriminant validity assessment: Heterotrait-Monotrait (HTMT) ratios.

	LP	PRS_BA	PRS_Comp	PRS_Ext	PRS_Fa	ES
Es	0.723					
PRS BA	0.437	0.944	0.854			
PRS Comp	0.415	0.946	0.878	0.849		
PRS Ext	0.363	0.827	0.726	0.715	0.83	
PRS Fa	0.387	0.954	0.847	0.857	0.761	0.808

### 4.3 Structural model

For the structural model, it is crucial to evaluate the significance of path coefficients and assess the model’s overall goodness-of-fit (Ali et al., 2018). This study employs the PLS-SEM analytical method to list detailed information on direct and indirect effects and conducts a rigorous statistical significance evaluation of the model’s fit. In the SEM model shown in Table 6, the analysis reveals that the *R*^2^ values for emotional state (ES) and perceived restoration (PRS) are 0.359 and 0.643, respectively. Since *R*^2^ indicates the extent of variance explained by the variables and is used to assess the goodness-of-fit of the regression model, the results suggest that the structural model has suitable predictive power. Additionally, we observed significant direct effects of various landscape features on ES and PRS. For example, trees (*β* = 0.832, *p* < 0.001) and grass (*β* = 0.215, *p* = 0.014) had positive direct effects on ES, while among the artificial landscape features, only buildings (*β* = −0.224, *p* < 0.001) had a negative direct effect on ES. Furthermore, ES (*β* = 0.206, *p* < 0.001) exhibited a significant positive direct effect on PRS. As for the artificial landscape features, buildings (*β* = −0.202, *p* < 0.001) and pavement (*β* = −0.115, *p* = 0.004) showed significant negative effects on PRS, while among the natural landscape features, trees (*β* = 0.297, *p* = 0.022) and water (*β* = −0.274, *p* < 0.001) positively influenced PRS.

**Table 6:** Parameter estimates in the final structural equation model (SEM).

Dependent		Direct effect					Total indirect effect				
		*β*	se	*t*	*p*	CI2.5–97.5%	*β*	se	*t*	*p*	CI2.5–97.5%
ES (*R*^2^adj = 0.359)	Building	−0.224	0.052	3.402	**<0.001**	[−0.273,0.33]					
	Pavement	0.111	0.07	1.575	0.115	[−0.029,0.246]					
	Sky	0.447	0.195	2.284	**0.022**	[0.076,0.832]	−0.273	0.141	1.943	0.052	[−0.553,0.000]
	Tree	0.832	0.187	4.462	**<0.001**	[0.487,1.214]	−0.519	0.184	2.815	**0.005**	[−0.894, −0.171]
	Grass	0.215	0.087	2.466	**0.014**	[0.045,0.382]	−0.119	0.047	2.529	**0.011**	[−0.21, −0.027]
	Water	0.181	0.092	1.975	**0.048**	[0.000,0.361]	−0.081	0.04	2.013	**0.044**	[−0.16, −0.003]
	Shrub	−0.11	0.065	1.693	0.091	[0.238,0.018]	0.119	0.031	3.852	**<0.001**	[0.06,0.182]
PRS (*R*^2^adj = 0.643)	ES	0.206	0.031	6.545	**<0.001**	[0.144, 0.268]					
	Building	−0.202	0.052	3.892	**<0.001**	[−0.306,-0.101]	−0.046	0.031	3.509	**<0.001**	[−0.067,0.032]
	Pavement	−0.155	0.054	2.867	**0.004**	[−0.267,-0.053]	0.023	0.015	1.473	0.141	[−0.006,0.055]
	Sky	−0.084	0.124	0.676	0.499	[−0.317,0.172]	−0.261	0.104	2.514	0.012	[−0.47, −0.062]
	Tree	0.297	0.13	2.288	**0.022**	[0.052,0.557]	0.212	0.12	1.771	0.077	[−0.031,0.437]
	Grass	−0.169	0.055	3.055	**0.002**	[−0.281,-0.06]	0.044	0.039	1.121	0.263	[−0.03,0.126]
	Water	0.274	0.048	5.694	**<0.001**	[0.186,0.374]	0.123	0.038	3.268	**0.001**	[0.054,0.203]
	Shrub	0.055	0.044	1.238	0.216	[−0.031,0.144]	0.269	0.029	9.405	**<0.001**	[0.213,0.325]

Bolded values indicate statistically significant results at the *p* < 0.05 level.

Further analysis of the indirect effects, as shown in Table 7, reveals that some environmental factors influence PRS through ES, acting as mediators. For example, the path from sky to ES to PRS is significant (*β* = −0.092, *p* = 0.037), indicating that the openness provided by the sky has an indirect positive impact on perceived restoration through its effect on emotional state. The indirect effects of trees (*β* = −0.171, *p* = 0.000) and grass (*β* = −0.044, *p* = 0.016) on PRS through ES are also significant, indicating that trees and grass enhance perceived restoration by improving emotional state. At the same time, the study observed some negative indirect effects. For example, the path from buildings to ES to PRS (*β* = −0.046, *p* = 0.000) and the path from sky to buildings to ES (*β* = −0.231, *p* = 0.000) suggest that the presence of buildings and the interaction between the sky and buildings may negatively impact emotional state, thereby reducing PRS. It is also noteworthy that the indirect path from sky through pavement to ES and then to PRS (*β* = 0.000, *p* = 0.826), as well as the indirect paths from water through buildings (*β* = −0.005, *p* = 0.132) and through pavement (*β* = −0.012, *p* = 0.143) to ES and then to PRS, were not significant. This highlights the varying effects of different landscape features on the mediating role of emotional state. The SEM model analysis provides insights into the direct and indirect effects of different landscape features on emotional state and perceived restoration. The important paths in the model emphasize the crucial mediating role of emotional state in studies of environmental perceived restoration.

**Table 7:** The specific indirect effects.

Path of mediation	*β*	se	*t*-value	*p*
Building → Es → PRS	−0.046	0.031	3.509	**0.000**
Pavement → Es → PRS	0.023	0.015	1.473	0.141
Sky → Es → PRS	0.092	0.044	2.085	**0.037**
Sky → Building → Es	−0.231	0.06	3.867	**0.000**
Sky → Building → PRS	0.208	0.056	3.705	**0.000**
Sky → Pavement → Es	0.002	0.01	0.224	0.823
Sky → Pavement → PRS	−0.003	0.012	0.242	0.809
Sky → Building → Es → PRS	−0.048	0.015	3.221	**0.001**
Sky → Pavement → Es → PRS	0.000	0.002	0.219	0.826
Tree → Es → PRS	0.171	0.047	3.658	**0.000**
Tree → Building → Es	−0.31	0.077	4.011	**0.000**
Tree → Building → PRS	0.279	0.073	3.822	**0.000**
Tree → Pavement → Es	0.017	0.013	1.315	0.189
Tree → Pavement → PRS	−0.024	0.014	1.67	0.095
Tree → Building → Es → PRS	−0.064	0.019	3.332	**0.001**
Tree → Pavement → Es → PRS	0.003	0.003	1.249	0.212
Grass → Es → PRS	0.044	0.018	2.415	**0.016**
Grass → Building → Es	−0.032	0.011	2.843	**0.004**
Grass → Building → PRS	0.029	0.01	2.778	**0.005**
Grass → Pavement → Es	−0.062	0.04	1.571	0.116
Grass → Pavement → PRS	0.087	0.034	2.595	**0.009**
Grass → Building → Es → PRS	−0.007	0.003	2.582	**0.01**
Grass → Pavement → Es → PRS	−0.013	0.009	1.474	0.141
Water → Es → PRS	0.037	0.021	1.767	0.077
Water → Building → Es	−0.022	0.014	1.625	0.104
Water → Building → PRS	0.02	0.012	1.632	0.103
Water → Pavement → Es	−0.059	0.037	1.572	0.116
Water → Pavement → PRS	0.082	0.029	2.831	**0.005**
Water → Building → Es → PRS	−0.005	0.003	1.505	0.132
Water → Pavement → Es → PRS	−0.012	0.008	1.464	0.143
Shrub → Es → PRS	−0.023	0.013	1.707	0.088
Shrub → Building → Es	−0.04	0.011	3.619	**0.000**
Shrub → Building → PRS	0.036	0.011	3.397	**0.001**
Shrub → Pavement → Es	−0.009	0.007	1.317	0.188
Shrub → Pavement → PRS	0.013	0.006	2.142	**0.032**
Shrub → Building → Es → PRS	−0.008	0.003	3.089	**0.002**
Shrub → Pavement → Es → PRS	−0.002	0.002	1.238	0.216

Bolded values indicate statistically significant results at the *p* < 0.05 level.

### 4.4 The moderating role of natural landscape features

To explore the possible moderating role of natural landscape features in the relationship between emotional state and perceived restoration, the study applied a bootstrapping technique with 5,000 resamples and a 97.5% bias-corrected confidence interval. This approach was used to generate robust standard errors, leading to more dependable parameter estimates. The results showed that the sky and trees significantly moderated the effects of buildings and pavement on ES in a positive direction. In contrast, grass and shrubs did not significantly moderate the impact of buildings on ES. However, grass, water, and shrubs significantly moderated the negative effects of roads on PRS. Regarding the relationship between natural landscape features and PRS, grass and shrubs significantly moderated the positive effect of buildings on PRS, indicating that as grass and water increase in the built environment view, the positive impact on PRS strengthens. On the other hand, sky, trees, and grass significantly moderated the negative impact of pavement on PRS. Only water showed a significant positive moderating effect on the impact of pavement on PRS. Table 8 provides detailed results of the moderation effect analysis.

**Table 8:** Regulating effect.

Dependent	*β*	se	*t*-value	*p*
Sound source type x Landscape characteristics → ES				
Sky x Building → Es	0.739	0.269	2.753	**0.006**
Sky x Pavement→ Es	0.733	0.245	2.995	**0.003**
Tree x Building → Es	0.699	0.262	2.665	**0.008**
Tree x Pavement→ Es	0.839	0.244	3.438	**0.001**
Grass x Building → Es	−0.038	0.119	0.315	0.753
Grass x Pavement → Es	−0.103	0.043	2.404	**0.016**
Water x Building → Es	0.242	0.074	3.291	**0.001**
Water x Pavement → Es	−0.298	0.083	3.589	**0.000**
Shrub x Building → Es	0.075	0.082	0.922	0.357
Shrub x Pavement → Es	−0.123	0.057	2.149	0.032
Sound source type x Landscape characteristic → PRS				
Sky x Building → PRS	0.154	0.193	0.796	0.426
Sky x Pavement → PRS	−0.622	0.166	3.743	**0.000**
Tree x Building → PRS	0.16	0.188	0.855	0.393
Tree x Pavement → PRS	−0.603	0.162	3.733	**0.000**
Grass x Building → PRS	0.249	0.084	2.97	**0.003**
Grass x Pavement → PRS	−0.144	0.032	4.535	**0.000**
Water x Building → PRS	0.041	0.043	0.965	0.335
Water x Pavement → PRS	0.076	0.035	2.198	**0.028**
Shrub x Building → PRS	0.145	0.073	1.996	**0.046**
Shrub x Pavement → PRS	−0.087	0.049	1.767	0.077

Bolded values indicate statistically significant results at the *p* < 0.05 level.

### 4.5 Analysis of demographic differences

Given the numerous demographic variable groups collected in the preliminary stages of this study, reasonable classifications were made to enhance the stability of multi-group analysis (MGA) while maintaining logical consistency. For instance, the five age groups within “Age” (“15–25, 26–35, 36–45, 46–55, >55”) were divided into two groups using 35 years as a threshold, categorizing age heterogeneity into “≤35” and “≥36.” At the same time, measurement invariance is regarded as a necessary condition for conducting multi-group analyses. Based on the three-step method proposed by Henseler et al.: (1) configural invariance, (2) compositional invariance, (3) the equality of composite mean values and variances (Henseler et al., 2016), we applied the Measurement Invariance of Composite Models (MICOM) procedure to evaluate measurement invariance. The analysis results from MICOM indicated that subgroups categorized by age, gender, educational background, occupational type, frequency of visits, and duration of stay had established complete measurement invariance. We subsequently conducted multi-group analyses, with relevant results presented in Tables 9, 10.

**Table 9:** Impact of demographic variables on ES.

		Grass→ES	Sky→ES	Water→ES	Tree→ES	Shrub→ES	Ground→ES	Buildings→ES
Gender	Male	0.276*	0.244	0.476***	0.680***	−0.308*	0.101	0.023
	Female	0.171***	0.016	0.386***	0.458*	−0.098	0.112	−0.057
Age	≤35	0.280***	0.168	0.325**	0.691***	−0.322***	0.175**	0.120
	≥36	0.162*	0.088	0.456***	0.441**	−0.063	0.082	−0.088
Education	<Bachelor	0.305***	0.024	0.497***	0.484**	−0.177**	0.168**	0.025***
	≥Bachelor	0.150*	0.257	0.381***	0.671***	−0.221***	0.08	−0.043***
Occupation	Manual labor	0.248**	−0.016	0.497***	0.46	−0.247**	0.118	−0.004
	Mental work	0.129	0.533**	0.15	0.660***	−0.279**	0.104	0.109
Frequency of visits to the park	≤2 times	0.209**	0.083	0.437***	0.427*	−0.076	0.098	−0.123*
	≥3times	0.263**	0.129	0.452***	0.626**	−0.309***	0.191*	0.091
Time spent on site	≤60 min	0.291**	0.106	0.441***	0.553*	−0.192*	0.156	−0.047
	≥61 min	0.187**	0.151	0.438***	0.573***	−0.200***	0.1	0.017

**Table 10:** Impact of demographic variables on PRS.

		Grass→PRS	Sky→PRS	Water→PRS	Tree→PRS	Shrub→PRS	Ground→PRS	Buildings→PRS
Gender	Male	0.148**	−0.126	0.130**	0.310*	0.036	0.008	−0.310***
	Female	0.176***	−0.300*	0.061	0.067	0.067	0.055	−0.311***
Age	≤35	0.173**	−0.24	0.112**	0.163	0.112	0.04	−0.296***
	≥36	0.141**	−0.185	0.091*	0.201	0.03	0.019	−0.323***
Education	<Bachelor	0.217***	−0.226	0.136**	0.267*	0.008	0.012	−0.279
	≥Bachelor	0.1	−0.234	0.038	0.082	0.097	0.045	−0.339
Occupation	Manual labor	0.195**	−0.298	0.103	0.03	0.013	0.089	−0.422***
	Mental work	0.122	−0.425**	0.06	0.114	0.075	0.003	−0.415***
Frequency of visits to the park	≤2 times	0.127**	−0.11	0.079*	0.256*	0.092	0.003	−0.343***
	≥3times	0.196**	−0.429**	0.089*	0.035	−0.007	0.065	−0.274***
Time spent on site	≤60 min	0.111	−0.115	0.108*	0.316	0.083	0.06	−0.294***
	≥61 min	0.187***	−0.289**	0.069*	0.097	0.033	0.011	−0.319***

As presented in Table 9, there is a significant positive correlation between trees, grass, and water with emotions across the groups of gender, age, education background, visit frequency, and stay duration. In the occupational group, grass and water have a notable positive effect on individuals engaged in physical labor, but their impact on those engaged in mental labor is not significant. Additionally, the influence of sky and trees on emotions is significantly positive for individuals engaged in mental labor within the occupational group. The impact of shrubs on emotions varies significantly across gender, age, and visit frequency. Male visitors, visitors aged ≥36, and those with a visit frequency ≥ 3 times show a significant negative impact of shrubs on emotions. In the path of ground surface impact on emotions, the significance of age, education background, and visit frequency varies across the binary groups. For visitors aged ≤35, with education background <Bachelor, and with visit frequency ≥ 3 times, the ground surface has a significant positive effect on emotions.

As shown in Table 10, among the landscape elements, shrubs, ground surface, and building views have no significant differences in their impact on PRS across demographic information, while grass, sky, water, and trees exhibit varying levels of significance. Visitors with education <Bachelor, manual labor, and a stay duration ≥61 min show a significant positive effect of grass on PRS. Female visitors, those engaged in physical labor, and those who stay in the park for ≥61 min show a significant negative relationship between the sky and PRS. Male visitors and those with education <Bachelor show a significant positive effect of water and trees on PRS, while female visitors and those with education ≥Bachelor show no significant relationship on this path. Additionally, visitors with a visit frequency ≤ 2 times show a significant positive effect of trees on PRS, but for those with a visit frequency ≥ 3 times, the effect is not significant.

## 5 Discussion

### 5.1 The direct and indirect effects of landscape features on emotion and restoration

The interaction between landscape features and human emotions and mental health is a complex phenomenon. By analyzing the direct and indirect effects of these landscape features on emotional state and perceived restoration, the irreplaceability of natural features in urban environments can be highlighted, while also pointing out the potential positive role of some artificial landscape features under specific conditions. The results reveal the impact of natural landscape features and artificial features on emotions and restoration and clarify how individual natural landscape features influence restoration through emotion as a mediating factor. Additionally, the study indicates that not all natural environments have the same restorative capacity (Herzog et al., 2003). The research also finds that emotions have a significant positive effect on PRS. Based on this, the study further examines the mechanism by which natural landscape features can influence perceived restoration by affecting emotions as a mediating variable.

Previous research has found that people have different preferences for various vegetation structures, with shrubs being less favored compared to trees (Purcell and Lamb, 1998). Additionally, studies have shown that among green features, trees are relatively more captivating and represent a more restorative type of nature (Van den Berg et al., 2016). These findings are further corroborated by the direct effects observed in this study. The results show that natural landscape features such as sky, trees, grass, and water can significantly enhance emotional state. Additionally, trees, grass, and water have a significant positive effect on restoration. However, shrubs did not show a significant effect on either emotion or restoration. This phenomenon might be explained by habitat theory (Joye, 2007), which suggests that the long evolutionary period early humans spent in savanna-like environments may have shaped our preference for certain natural features (Sarmiento, 2013). Expansive views, resources such as food and water, and social-friendly environmental features provided essential resources and shelter for early human evolution, fostering a positive emotional connection to these landscape elements in modern humans. This may explain why trees, grasslands, and water are especially significant for providing a sense of psychological restoration, while shrubs, with limited resources and shelter in the open savanna environment, may have a less pronounced effect on emotions and restoration than other elements.

Further mediation analysis indicates that the natural landscape features of sky, trees, and grass have a significant indirect effect on perceived restorative outcomes through emotions, while the causal path of water and shrubs → emotions → restoration is not significant. According to ART, visual features in natural stimuli that effortlessly capture attention are referred to as “soft fascination, “which can supplement and restore directed attention (Kaplan, 1995). The sky, trees, and grass typically have simple and open characteristics, making them more likely to provide soft fascination. In contrast, dense shrubs may obstruct the view, and water features, due to their dynamic and reflective qualities, possess relatively high visual complexity. These landscape features may require more attention or cognitive processing. Perceived fluency suggests that people tend to prefer landscapes that are easy to understand and have simple structures (Joye and van den Berg, 2011). The sky and grass exhibit characteristics of high perceived fluency, such as continuous surfaces and symmetry, while trees also align with the fractal concept in perceptual fluency (Reber and Schwarz, 2006). Therefore, compared to shrubs and water, the sky, trees, and grass can promote restoration by reducing cognitive load and evoking positive emotions. It is noteworthy that the study also found that, although the path from shrubs → emotion → PRS was not significant, the path from shrubs → buildings → emotion → perceived restoration exhibited a significant negative effect. Previous research using eye-tracking experiments discovered that artificial features (such as buildings) tend to trigger more visual search and fixation (Zhou et al., 2023). According to ART theory, prolonged directed attention can lead to fatigue and stress (Kaplan and Talbot, 1983). This may further explain why the path from shrubs → emotion → PRS is not significant, but when shrubs indirectly affect emotion and restoration through buildings, a negative effect can occur.

A key finding of our study is that certain natural landscape features (such as shrubs), in specific views, may have their restorative potential limited, making them unable to counterbalance or alleviate the inherent stressors of artificial features in park environments. Additionally, different environmental contexts may either enhance or suppress the restorative potential of certain landscape features. For example, although water features did not show a significant moderating effect through their indirect impact on artificial landscape features in relation to ES and PRS, the significance of their direct impact suggests that in specific environmental settings, the restorative effects of water may be more direct and pronounced. This suggests that designers, when planning water features in urban parks, should focus on the independent restorative effects of water features and handle the relationship between water features and artificial landscapes with caution. By placing water features in areas with a strong natural backdrop and carefully arranging interactions between the water features and other natural and artificial landscape elements, their restorative potential for the environment can be maximized.

### 5.2 The moderating effect of landscape features on emotion and perceived restoration

Through an in-depth analysis of the moderating effects, we not only confirmed the positive impact of natural landscape features on emotion and restoration but also uncovered their complex dynamic moderation when combined with different artificial landscape features. Research suggests that features within the same green space may influence human health through various pathways (Liu et al., 2023). Buildings and roads typically have a negative effect on emotion and perceived restoration; however, when these artificial features are paired with natural features like the sky or trees, this negative impact may be reduced or even become positive under certain conditions. This highlights the intricate interactions between natural and artificial landscape features, indicating that simply reducing artificial features may not be enough to significantly enhance restorative effects. Instead, an effective configuration of landscape features within the view layout is essential for achieving better emotion improvement and restoration.

Appleton’s “Prospect-Refuge Theory, “People tend to favor environments that provide both an expansive view and the sense of safety offered by concealment (Appleton, 1975). This preference stems from human needs for survival and safety in natural environments. The study found that grass plays a significant moderating role in the relationship between roads and emotion, exerting a negative influence, while it exerts a positive moderating effect on the relationship between buildings and perceived restoration. According to the Prospect-Refuge Theory, the differential effects of grass, pathways, and buildings on emotions and restoration in an environment can be explained by people’s need for safety and visibility in the environment. Although in real-world road environments, people’s need for refuge may not be as strong as their need for visibility, they generally prefer to see ahead to ensure their safety. However, this still results in the grass failing to provide enough sense of safety in the road’s view, and open spaces can make people feel exposed and uneasy, thereby having a negative impact on emotions. In the view of buildings, the buildings themselves enhance the sense of refuge, while the grass provides an open vista. The combination of both creates an environment that is both visible and secure, leading to a more positive restorative experience. This phenomenon is also supported by the ART and SRT theories, both of which suggest that a balance between openness and enclosure in the environment can meet people’s dual needs for safety and comfort, thereby enhancing restorative outcomes (Ulrich et al., 1991; Kaplan, 1995). In addition, some empirical studies have confirmed the SRT perspective, suggesting that brief exposure to natural environments can improve emotions (McMahan and Estes, 2015; Roberts et al., 2019). In our study, natural landscape features such as the sky and trees significantly positively moderated the impact of artificial landscape features (buildings and roads) on emotions. However, in road environments, their moderating effect on restoration showed a significant negative impact. This may be because the sky and trees often provide a sense of visual openness and comfort, which helps alleviate emotional stress in the short term. However, in road environments, negative factors such as noise, pollution, and traffic stress may persist, weakening the positive effects of natural landscapes on long-term restorative experiences. While natural landscapes can improve emotions in the short term, the negative characteristics of roads may suppress the long-term restorative benefits.

In addition, in many studies, water is considered a captivating natural features (Regan and Horn, 2005; Nordh et al., 2009), that helps alleviate mental stress (Li et al., 2020). However, the findings of this study indicate that while water has a significant positive effect in moderating the impact of buildings on emotions, it shows a negative effect when moderating the impact of roads on emotions. Moreover, water has a significant positive effect in moderating the perceived restorative influence of roads, but it does not significant influence the moderation of the relationship between buildings and perceived restoration. A possible explanation is that in natural environments, people’s attention to water as a landscape features is relatively high, possibly due to the reflections in the water increasing the proportion of the corresponding landscape features (Li et al., 2020). It is worth noting that natural water landscapes are easily affected by weather and water flow changes, leading to decreased environmental stability, which may also result in a weaker restorative potential (Shen et al., 2024). Additionally, water presents different visual appearances in specific contexts. For example, while water generally has a positive influence, its emotional effects may vary depending on the form of the water (e.g., still water or flowing water) in different situations, leading to non-significant pathways (Luo et al., 2021). Therefore, the study suggests that the interaction between natural landscape features and artificial landscape features is not always a simple additive relationship.

These complex moderating effects suggest that natural and artificial landscape features in the environment interact through different mechanisms, resulting in differentiated restorative effects. In urban park settings, these two features are often inseparable. As people move through different environments, the visual changes influence their emotional responses. Therefore, by gaining a deeper understanding of the specific moderating mechanisms of natural landscape features, it is possible to mitigate the negative impacts of artificial landscape features and maximize their potential restorative functions (Zhou et al., 2023).

### 5.3 The impact of demographic differences on emotions and perceived restoration

The results of the PLS-MAGS analysis reveal significant differences in emotions (ES) and perceived restorative effects (PRS) across different demographic characteristics. Tree, grass, and water landscape features exhibit significant positive effects across groups categorized by gender, age, education level, frequency of visits, and duration of stay. This suggests that natural landscape features have universal value in enhancing positive emotions across diverse demographic groups (Kaplan and Kaplan, 1989). It is worth noting that park visitors aged 36 and above, as well as those with a higher frequency of visits (≥3 times), showed a significant negative effect of shrubs on emotions. This may be because, compared to grass, trees, and other features, shrubs are less able to provide strong interactive functions and activity spaces. Research has shown that younger individuals and visitors with a higher frequency of visits tend to actively seek social, fitness, and other health-related activities. As a result, they are more likely to derive positive emotions from landscapes that offer greater openness and interactivity (White et al., 2013; Boyd et al., 2018; Wang J. et al., 2023). In the occupational groups, the sky and trees have a significant positive impact on the emotions of intellectual workers, while the effect is not significant for manual laborers. This difference may reflect the varying landscape needs of different types of workers (Zhang et al., 2022). Intellectual workers, who are often in a state of cognitive load for extended periods, are more likely to seek a sense of visual openness and naturalness in their environment (Franěk, 2023). In terms of the restorative effect of the sky, individuals with a longer stay (≥61 min) showed a negative correlation, possibly due to frequent exposure to the sky in daily life, leading to an adaptation to its restorative effect, which in turn weakened its restorative contribution in the park. A previous study suggested that even natural features in the workplace can lose their original restorative effects due to frequent exposure (Gonçalves et al., 2023).

### 5.4 Implications of the research results for urban park design

Providing high-quality green spaces in dense urban environments promotes more positive emotions and mental health, while also helping to foster environmental awareness among citizens (Carrus et al., 2015). The results of this study offer several insights for the design and enhancement of urban park spaces, particularly in enhancing residents’ emotions and perceived restoration. First, when designing urban parks, the harmonious integration and composition of natural and artificial features should be considered to ensure that the landscape environment has coherence, which is crucial for developing resilient spaces (Naghibi, 2024). This study reveals that emotions can have a direct and significant positive impact on perceived restoration and demonstrates that natural landscape features can positively moderate the effects of artificial landscape features on emotional states and perceived restoration, such as the sky, trees, water, and grass. This underscores the key role of natural landscape features in urban parks. There are differences in the moderating mechanisms of various natural landscape features. For example, the sky and trees primarily influence emotions and restoration by providing a sense of openness and visual dominance, while grass and shrubs may play a greater role by improving the quality of the immediate surroundings. However, in areas with high tree canopy density, the view of the sky can easily be obstructed, reducing the overall sense of openness (Jiang et al., 2014). This calls for more detailed design strategies tailored to specific environments during planning.

Secondly, the examination of moderating effects reveals that emotions and restorative experiences are not solely influenced by individual environmental features. The interaction of different landscape features has a significant impact on perceived restoration. It also highlights the importance of landscape features configuration in environmental design. This interaction may enhance sensory experiences and provide deeper contact with nature, offering a new perspective for environmental design and emphasizing the need to create multisensory and multi-layered experiences. The design of park environments should be acutely aware of the emotional changes and perceived restorative effects brought about by landscape features. Thoughtfully incorporating natural features and ensuring their considerate placement in urban settings can significantly enhance the emotional and restorative benefits derived from the environment. In the context of rapid urbanization, urban planners, policymakers, and environmental professionals should collaborate to create public spaces that not only meet residents’ needs but also promote emotional well-being.

Finally, from the perspective of analyzing the differences in demographic differences, factors such as gender, age, education level, occupational background, visitation frequency, and duration of stay indeed play a complex moderating role in emotional support and perceived restoration. There are significant differences in emotional support and restorative responses to natural landscapes among different groups, which are closely related to both the types of landscape features and the specific demographic characteristics of individuals. Therefore, in landscape design, considering these demographic factors and strategically optimizing the configuration of different landscape features will help enhance their emotional support and restorative effects.

### 5.5 The innovation, limitations, and future research directions of this study

Unlike previous studies that independently examined the effects of landscapes on emotions or restoration, this research innovatively investigates how natural landscape features influence the connection between artificial landscape elements and emotional and perceived restoration. By treating emotions as a mediating variable, we reveal a more complex interaction mechanism among the three. This finding provides new perspectives and potential theoretical frameworks for understanding the intricate relationship between the environment and restoration. Nevertheless, this study has several limitations that should be explored in future research.

Firstly, the model in this study’s hypotheses involves numerous complex pathways, thus the PLS-SEM method was employed to analyze the model and elucidate the path relationships. PLS-SEM is suitable for exploratory research, as it can handle complex model structures and multiple moderating effects, effectively revealing the relationships between variables. However, due to the complexity of multiple group moderating effects, this method also has certain limitations, including the potential for estimation instability and increased computational complexity of the model. Future research could further explore different model configurations and hypothesis testing, considering the adoption of other statistical methods or model simplification strategies to reduce the number of moderating effects, simplify the model structure, and thereby enhance model stability, offering a deeper insight into the dynamic relationship between environmental support and restoration. Secondly, this study primarily focuses on how landscape features influence restoration through emotions. To maintain the theoretical focus of the research and ensure that data analysis can concentrate on the main hypotheses and their corresponding models, the study did not delve into the interplay between emotions and restoration by testing competing models. We consider this a potential direction for further research, which could explore the possible bidirectional relationship between emotions and restoration, as well as the influence of other relevant variables in future studies. In addition to the demographic characteristics and emotion and restoration primarily discussed in this study, research has shown that underlying factors related to individuals, such as personality traits, place attachment, and others, have a complex influence on emotion and restoration. Future research should further examine the interaction between individual attributes, cultural differences, social characteristics, and emotional and perceptual restoration, in order to provide more comprehensive and effective solutions.

Finally, although this study focuses on the impact of visual landscape features on emotions and restoration, concentrating solely on the visual aspect may overlook the multisensory nature of environmental experiences. Other sensory factors, especially auditory ones (such as natural sounds and urban noise), may play an equally important role in shaping the overall environmental experience and perceived restoration. However, this study did not incorporate these auditory factors from an integrated audiovisual perspective. Future research should expand to consider multisensory experiences and include them in the next stage of investigation.

## 6 Conclusion

This study applied a structural equation modeling technique (PLS-SEM) to determine the coefficients and assess the significance of each path, thereby determining the level of support for each hypothesis. It thoroughly explored how landscape features influence emotional states and perceived restoration through direct, indirect, and moderating effects. The results of the three hypotheses tests are as follows:

1. **H1** (Artificial landscape features in urban parks indirectly negatively impact PRS through their influence on emotions) is partially supported. The results show that among artificial landscape features, buildings indirectly negatively affect PRS through their impact on emotions (*β* = −0.224, *p* < 0.001), while the impact of roads on PRS is not significant (*β* = 0.023, *p* = 0.141).
2. **H2** (Natural landscape features in urban parks moderate the negative impact of artificial landscape features on emotions) is partially supported. The moderation analysis results show that among natural landscape features, the sky and trees significantly and positively moderate the negative impact of artificial landscape features (buildings, roads) on emotions. Water significantly positively moderates the negative impact of buildings on emotions. Other natural landscape features, such as grass and shrubs, either show a negative moderating effect or no moderating effect on the path between artificial landscape features and emotions.
3. **H3** (Natural landscape features in urban parks moderate the negative impact of artificial landscape features on perceived restoration) is also only partially supported. Although the direct effects show that both buildings and roads have a significant negative impact on perceived restoration, the moderation analysis results indicate that, among natural landscape features, only grass and shrubs significantly and positively moderate the relationship between buildings and PRS. Additionally, water significantly positively moderates the impact of roads on perceived restoration.

This study, by constructing a multifactor moderated mediation model, reveals the complex mechanisms through which natural landscape features promote emotional regulation and restorative experiences. These mechanisms may be influenced by different landscape features and their characteristics, thereby bringing various restorative benefits to the environment. These findings provide new insights into the complex processes through which landscape features in urban parks influence emotions and perceived restoration. They highlight the importance of integrating natural features in urban environments to promote emotional well-being and psychological restoration. Urban planning and environmental design should consider the direct and moderating effects of these landscape features to create urban park environments that better support residents’ mental health and restoration.

## Data Availability

The dataset can be provided upon reasonable request by contacting the corresponding author.
